# Immunotherapy in NSCLC Patients with Brain Metastases

**DOI:** 10.3390/ijms23137068

**Published:** 2022-06-25

**Authors:** Silvia Buriolla, Giacomo Pelizzari, Carla Corvaja, Martina Alberti, Giada Targato, Martina Bortolot, Sara Torresan, Francesco Cortiula, Gianpiero Fasola, Alessandro Follador

**Affiliations:** 1 Department of Medicine (DAME), University of Udine, 33100 Udine, Italy; silviaburiolla@outlook.it (S.B.); carlacorvaja@gmail.com (C.C.); martiz.alberti93@gmail.com (M.A.); giadatargato@gmail.com (G.T.); bortolot.martina@spes.uniud.it (M.B.); torresan.sara@spes.uniud.it (S.T.); 2 Department of Oncology, Azienda Sanitaria Universitaria Friuli Centrale (ASUFC), 33100 Udine, Italy; francesco.cortiula@asufc.sanita.fvg.it (F.C.); gianpiero.fasola@asufc.sanita.fvg.it (G.F.); alessandro.follador@asufc.sanita.fvg.it (A.F.)

**Keywords:** NSCLC, immunotherapy, brain, metastases

## Abstract

Approximately 40% of unselected non-small cell lung cancer (NSCLC) patients develop brain metastases (BMs) during their disease, with considerable morbidity and mortality. The management of BMs in patients with NSCLC is a clinical challenge and requires a multidisciplinary approach to gain effective intracranial disease control. Over the last decade, immune checkpoint inhibitors (ICIs) have emerged as a game-changer in the treatment landscape of advanced NSCLC, with significant improvements in survival outcomes, although patients with BMs are mostly underrepresented in randomized clinical trials. Moreover, the safety and activity of ICIs and radiotherapy combinations compared with single-agent or sequential modalities is still under evaluation to establish the optimal management of these patients. The aim of this review is to summarize the state-of-the-art of clinical evidence of ICIs intracranial activity and the main challenges of incorporating these agents in the treatment armamentarium of NSCLC patients with BMs.

## 1. Introduction

Approximately 16–60% of non-small cell lung cancer (NSCLC) patients develop brain metastases (BMs), both in oncogene-addicted disease and in patients that do not harbor actionable mutations [1,2]. The management of BMs is a clinical challenge and requires a multidisciplinary approach to provide prompt local control. However, the survival benefit of conventional therapies (e.g., surgery, radiotherapy, and palliation with corticosteroids) is only marginal and worsened by a high incidence of neurotoxicity that often leads to delayed or compromised systemic therapy and high mortality rates [3,4,5,6]. Notably, the improvement of neuro-imaging techniques and the prolonged survival observed in NSCLC patients treated with novel systemic therapies increased the lifetime incidence of BMs in these patients [7]. Local strategies might be considered for the treatment of BMs, including stereotactic radiosurgery (SRS) alone or after surgical resection, and whole-brain radiotherapy (WBRT) in selected cases [8], although neurocognitive toxicities represent a critical limitation. Over the last decade, a more profound knowledge of the mutual interplay between tumor cells and the tumor microenvironment, a critical regulator of immune escape, has led to the development of immune checkpoint inhibitors (ICIs) that target CTLA-4 or PD-1 on exhausted CD8+ T-cells, or PD-L1 on tumor cells, to restore T-cells’ anti-tumor activity [9]. Indeed, ICIs have emerged as a game-changer in the treatment landscape of advanced NSCLC, significantly improving survival outcomes [10,11,12,13,14,15]. The peculiar immunological environment of the brain is challenging and the question of whether ICIs’ efficacy in BMs is impaired needs to be prospectively elucidated [16]. Patients with BMs have been systematically underrepresented in pivotal ICI clinical trials, due to concerns about the ability of monoclonal antibodies to penetrate the blood–brain barrier (BBB), the potential negative impact of concomitant steroids, and the risk of pseudoprogression [17]. Preliminary data from single-arm phase 1–2 trials, post hoc analyses, and retrospective series have demonstrated durable intracranial activity, extracranial responses, and improved survival outcomes with ICIs regardless of the presence of baseline BMs [15]. Indeed, although the CNS is isolated by the BBB (consisting of endothelial cells, pericytes, and the foot processes of astrocytes), in patients with BMs the disruption of BBB integrity favors drugs penetration and the infiltration of peripheral activated T-cells [18].

However encouraging, these results represent highly selected subgroups of patients with small, asymptomatic, or previously treated BMs, and their applicability to the broader population of NSCLC patients is limited. Moreover, it is crucial to evaluate the safety and activity of ICI and radiotherapy combinations compared with single-agent modality and establish the optimal management of patients with CNS pseudoprogression or oligoprogression [19]. Furthermore, PD-L1 expression across the CNS and extracranial disease and its predictive value for ICIs’ efficacy in BMs warrant further investigation [20,21]. Finally, it is critical to standardize intracranial disease response criteria for prospective clinical trials.

The aim of this review is to summarize the state-of-the-art of clinical evidence for ICIs’ intracranial activity and outline the main challenges of incorporating these agents in the treatment armamentarium of NSCLC patients with BMs.

## 2. Single-Agent Anti-PD-L1/PD-1 or Anti-CTLA-4 Monoclonal Antibodies

The first immune checkpoint inhibitor that showed efficacy in treating brain metastases was the anti-CTLA-4 monoclonal antibody ipilimumab. Its activity and efficacy were investigated in a phase II study that enrolled patients with melanoma brain metastases: after 12 weeks, CNS disease control was achieved in 12 patients (24%, 13–38) with asymptomatic brain metastases vs. two patients (10%, 1–30) neurologically symptomatic on corticosteroid therapy, showing more efficacy in patients with indolent CNS disease [22]. Moreover, two expanded access programs in the USA and Italy showed the efficacy of ipilimumab in treating patients with asymptomatic brain metastases from melanoma, with a 1-year OS rate of 20% in both studies [23,24].

In NSCLC, the activity of ipilimumab was investigated in association with nivolumab, demonstrating an impressive CNS response. The first phase II trial which examined the activity of pembrolizumab, a PD-1 inhibitor antibody, in NSCLC patients with untreated or progressing brain metastases showed a CNS response in the cohort with PD-L1 expression ≥1% (29.7%; 15.9–47.0). Conversely, PD-L1 expression of less than 1% was associated with no response [25]. A pooled analysis of KEYNOTE-001, 010, 024, and 042 highlighted a higher activity of pembrolizumab compared to chemotherapy in patients with PD-L1 TPS ≥1% and TPS ≥50%, both with and without brain metastases. Among patients with brain metastases and PD-L1 TPS ≥50%, the HR was 0.67 (95% CI: 0.44–1.02) for OS and 0.70 (95% CI: 0.47–1.03) for PFS, with an ORR of 33.9% vs. 14.6% with chemotherapy. Moreover, a similar benefit was achieved in the PD-L1 TPS ≥1%, with an HR of 0.83 (95% CI: 0.62–1.10) for OS and 0.96 (95% CI: 0.73–1.25) for PFS, with an ORR of 26.1% vs. 18.1% in the chemotherapy group [26]. In addition, many retrospective trials support the efficiency of pembrolizumab in patients with brain metastases, showing similar results compared to patients without CNS disease. Furthermore, better responses in terms of OS, PFS, and ORR were detected in the PD-L1 TPS ≥50% cohorts [27,28].

Nivolumab, a PD-1 inhibitor monoclonal antibody, was compared with docetaxel in CheckMate 017 and 057 trials, which included previously treated squamous or non-squamous NSCLC patients, respectively. A pooled analysis of these two studies demonstrated a benefit of nivolumab, with a median OS of 7.6 vs. 6.2 months with docetaxel (HR 0.81) [29]. Various studies based on the nivolumab Italian expanded access program focused on the subgroup of patients with brain metastases, showing similar efficacy of nivolumab compared with the overall population, with a 1-year OS rate of 43% [30,31,32,33]. Analogous results were reported in the real-world UNIVOC study, which reported a 2-year OS rate of 28.5% (26.4–30.8) in patients with brain metastases compared with 30.0% (29.0–31.1) in those without [34]. Likewise, data from a real-world pooled analysis of NSCLC patients from Canada, France, and Germany treated with nivolumab did not show significant differences in terms of OS, with a 1-year OS rate of 44% in the subgroup of brain metastases vs. 50% in that without CNS involvement [35].

Atezolizumab, an anti-PD-L1 monoclonal antibody, was studied in patients with brain metastases and previously treated NSCLC patients in the OAK trial and demonstrated a benefit of atezolizumab compared with docetaxel in the overall population, regardless of history of asymptomatic, treated brain metastases. Particularly, in patients with asymptomatic, treated brain metastases, OS was 26.6% (95% CI: 15.1–38.1) in the atezolizumab arm and 19.3% (95% CI: 8.2–30.4) in the docetaxel arm at 24 months. In those without asymptomatic, treated brain metastases, OS was 31.6% (95% CI: 26.7–36.5) and 21.4% (95% CI: 16.9–25.9), respectively, at the same timepoint [36]. Consistent results were obtained in the phase II FIR trial [37].

Durvalumab, an anti-PD-L1 monoclonal antibody, was studied in the phase III PACIFIC trial and showed prolonged PFS in patients with stage III unresectable NSCLC after concurrent chemo-radiation treatment. In the updated analyses of the PACIFIC trial it emerged that patients treated with durvalumab had a lower incidence of new brain metastases than a placebo group (6.3% vs. 11.8%) [38].

Lastly, cemiplimab, a new anti-PD-1 monoclonal antibody whose efficacy was tested in the EMPOWER-Lung 1 trial in patients with PDL1 ≥50%, demonstrated a significant improvement in PFS and OS compared with platinum-based chemotherapy in the first-line setting (HR 0.45 and 0.17 respectively) [39].

Outcomes of patients with NSCLC and BM treated with ICI monotherapy are reported in Table 1.

## 3. Combination Therapy

### 3.1. Anti-PD-1 and Anti-CTLA-4 Therapy

Preclinical evidence shows that combination immunotherapy, through the increase in T-cell infiltration and the reduction of regulatory T-cells, may overcome the acquired resistance of ICI monotherapy, which could be developed as a result of alternative and compensatory immune checkpoints [41,42,43]. Nevertheless, data about ICI combinations in NSCLC patients with BM are limited.

In the CheckMate 227 trial (part 1a) 720 patients with PD-L1 expression >1% were enrolled and randomized to ICI combination (nivolumab plus ipilimumab) or histology-driven chemotherapy [44]. Eighty-one patients had asymptomatic or efficaciously treated BM and those treated with nivolumab plus ipilimumab showed longer overall survival compared to the chemotherapy group, with a hazard ratio for death of 0.68 (16.8 months vs. 13.4 months, respectively). Notably, similar data were obtained in the non-BM population strengthening the efficacy of ICI combinations in BM patients. A post hoc analysis confirmed the benefit of the combination therapy with an advantage in PFS (1y PFS 38% vs. 21%, HR 0.79), ORR (33% vs. 26%) and duration of response (mDOR 24.9 m vs. 8.4 m) [45]. Intriguingly, 46% of patients with baseline BM showed any-grade neurological adverse events compared to 42% of those treated with chemotherapy. To note, patients requiring more than 10 mg of prednisone or equivalent were excluded. Patients with NSCLC and BMs were also included in the Checkmate 817 trial investigating the safety and efficacy of flat-dose nivolumab plus weight-based low-dose ipilimumab. The study comprised a cohort of a special population (PS ECOG 2, HIV, hepatic or kidney impairment, brain metastasis) with 44 patients with BMs. Overall, the ORR was 37%, with a median PFS of 4.2 months [46].

### 3.2. ICIs and Chemotherapy

Historically, chemotherapy has had limited efficacy in the treatment of brain metastasis. However, chemotherapy has a recognized role in enhancing the efficacy of ICI due to the increase in neoantigens expression, the induction of immunogenic cell death, the upregulation of PD-L1 expression in the tumor microenvironment and the stimulation of T-cell response [47,48,49]. Based on this rationale, Powell et al. conducted a pooled analysis of Keynote-021 (cohort G), 189, and 407 trials investigating the efficacy of combining pembrolizumab and chemotherapy in BM patients [50]. Of the 1298 patients included, 171 (12.3% of the total population) had asymptomatic or stable pre-treated (Keynote-021) or not-pre-treated (Keynote 189 and 407) brain metastasis, and 105 were randomized to the immune-chemotherapy combination. Similarly to the non-BM group, pembrolizumab plus chemotherapy demonstrated better OS (18.8 months vs. 7.6 months, HR 0.48), PFS (6.9 months vs. 4.1 months, HR 0.44), ORR (39% vs. 17.7%), and DOR (11.3 months vs. 6.8 months), with a benefit maintained across all PD-L1 expression subgroups. These data were successively confirmed by the updated results of the Keynote 189 trial [51].

More recently, promising results were shown in the CheckMate 9LA trial testing the efficacy of nivolumab and ipilimumab in combination with histology-based chemotherapy for two cycles [52]. Of the 361 patients in the combination arm, 51 had pre-treated, stable, or asymptomatic brain metastasis and derived benefit from immuno-chemotherapy with a considerable gain in PFS (1-year PFS rate: 36% vs. 8%, mPFS: 10.6 vs. 4.1 months) and OS (1-year OS rate: 67% vs. 26%, mOS: 19.3 vs. 6.8 months) compared to patients treated with chemotherapy.

### 3.3. ICIs and Radiotherapy

Radiation therapy is often part of standard care to increase the local control of brain metastasis. Considering the lack of survival benefit and the risk of neurocognitive impairment, WBRT has progressively been replaced by SRS. Several pieces of evidence support the immunogenic role of radiotherapy through the modulation of antigen expression in tumor cells, the stimulation of IFN production, and the induction of immunogenic cell death. Basically, radiation therapy establishes an inflamed microenvironment and promotes T-cell trafficking from the periphery to the tumor [53,54,55,56].

If the synergy of SRS and immunotherapy is unanimously accepted, the optimal sequence is still to be defined. In this scenario, Schapira et al. conducted a retrospective study evaluating the combination of SRS and anti-PD-1 immunotherapy with regards to the timing of administration (concurrent treatment, prior to or after immunotherapy) [57]. The results showed the superiority of a concomitant treatment in terms of both OS (1-year OS 87.3% vs. 70.0% vs. 0%, *p* = 0.008) and distant brain failure (1-year DBF 38.5% vs. 65.8% vs. 100%, *p* = 0.042). To note, concomitant treatment was defined as the administration of SRS within one month of the last anti-PD-1 dose, according to the median half-life of the drugs. Similarly, Kotecha et al. demonstrated a benefit of concomitant SRS-immunotherapy in intracranial response rate (73% vs. 53%, *p* = 0.00) with an earlier response [58]. Furthermore, the OS was higher with a lower dose of steroids (mOS 25.1 months for no steroids vs. 10.20 months for ≤60 mg dexamethasone, *p* = 0.002). In some retrospective studies, the concomitant treatment also seemed to increase the local control compared to sequential SRS plus ICIs [59], albeit with controversial results and not always statistically significant [60,61,62].

Therefore, the combination of radiotherapy and ICI is reasonable and supported by a robust preclinical rationale, but more prospective studies are required.

### 3.4. ICIs and Antiangiogenic Drugs

Angiogenesis has an essential role in the modulation of growth, invasion, and metastatization and antiangiogenic drugs have been widely studied in patients with solid tumors. Moreover, bevacizumab may exert an immunogenic activity triggering T-reg proliferation and increasing the T-cells’ infiltration [63,64,65,66]. Based on this rationale, the combination of antiangiogenic drugs and ICI was tested.

The Impower 150 phase III randomized trial tested the combination of atezolizumab, bevacizumab, and chemotherapy (arm B) compared to atezolizumab and chemotherapy (arm A) and bevacizumab plus chemotherapy (group C) [11]. Even though data about BM patients were not presented, an exploratory analysis showed a lower rate of new BM in arm B (7% vs. 11.9% in arm A and 6% in arm C), suggesting that the combination of bevacizumab and atezolizumab could delay the onset of new brain lesions [67]. Similarly, the combination of nivolumab, bevacizumab, and chemotherapy were tested in the TASUKI-52 trial and compared to bevacizumab and chemotherapy [68]. In this study, 36 of the 275 patients (13%) in the experimental arm presented with stable, asymptomatic or pre-treated brain metastasis. This group showed a gain in PFS with a HR of 0.65 (10.5 months vs. 7.1 months), regardless of PD-L1 expression.

### 3.5. ICIs and Target Agents

There is a lack of evidence about combining immunotherapy and target agents for patients with an oncogene-addicted NSCLC. The presence of driver mutations dichotomously influences the treatment algorithm, as suggested by international guidelines [5,6,7]. Stage IV NSCLC patients harboring targetable mutations, in fact, should be treated with a first-line target therapy and are excluded from studies testing immunotherapy combinations, following the rationale of “the target first”. Interestingly, early results from studies investigating the combination of pembrolizumab and lenvatinib, a multi-tyrosine kinase inhibitor, have shown improved efficacy and response durability in several advanced solid tumors, including preliminary data for patients with treatment-refractory NSCLC [69]. More recently, promising results were also presented for the combination of pembrolizumab and ramucirumab, a VEGFR-2 antibody, in pre-treated patients with NSCLC, although the subgroup of patients with BMs was not explicitly evaluated [70]. Outcomes of patients with NSCLC and BMs treated with combination therapies are reported in Table 2.

## 4. Future Perspective and Ongoing Trials

The present review focuses on the complex therapeutic scenario of patients with BM NSCLC. In this setting, immune-checkpoint inhibitors demonstrated a significant benefit compared with standard treatment in terms of OS, PFS, and ORR. However, treatment with ICIs is not exempted from adverse events when ICIs are used in combination with chemotherapy, radiotherapy, or other types of drugs (e.g., bevacizumab or other ICIs) [71]. Cabanie et al. highlighted that brain radionecrosis is the main complication of radiotherapy combined with ICIs, occurring in 9.7% of the population, with 5% of patients experiencing grade 3 intracranial hypertension [72]. The CheckMate 9LA trial evidenced that the most common adverse events of any grade in the population treated with the combination of two ICIs (nivolumab and ipilimumab) and chemotherapy are diarrhea (3%), anemia (2%), and febrile neutropenia (3%). Interestingly, the most common adverse events for grade 1 were ICI-related (e.g., rash, hypothyroidism) [73]. Sugawara et al. investigated the combination of nivolumab, bevacizumab, and chemotherapy in their trial: the most common adverse events of grade 3 or 4 in the experimental arm were febrile neutropenia (15%), hypertension (13.6%) and rash (12.5%) [68].

Patients with BMs are mostly underrepresented in clinical trials [74] and prospective data regarding their treatment are limited to clinically selected subgroup of patients with asymptomatic and stable BMs, not requiring significative corticosteroid therapy. To provide real-world evidence on the outcome of these patients, an international real-world study evaluated outcomes of patients with NSCLC treated with first-line anti-PD-1 or anti-PD-L1 monotherapy, also including those with active BMs (39.2%), symptomatic BMs (14.3%), or receiving steroids (27.4%) [75]. In this retrospective series, patients with BMs showed shortened PFS (1.7 vs. 2.1 months, *p* = 0.009) and OS (8.6 months vs. 11.4 months, *p* = 0.035) compared to those without BMs. Moreover, active BMs and corticosteroids were negative prognostic factors in the BM subgroup. Therefore, this latter group of patients should be carefully evaluated at baseline, considering multimodal approaches, including radiotherapy and/or chemo-immunotherapy combinations. Interestingly, the Atezo-Brain phase II trial explored the activity of atezolizumab associated with chemotherapy as first-line treatment in patients with non-squamous NSCLC and asymptomatic and untreated BMs (anticonvulsants and dexamethasone ≤4 mg were allowed) [40]. The study included 40 patients (42.5% were receiving steroids): the observed intracranial and systemic PFS were 7.1 months and 8.9 months, respectively, with an intracranial ORR of 42.5%.

Currently, several ongoing studies with ICIs include patients with NSCLC and BMs. For example, the NCT05012254 trial explores the efficacy of nivolumab plus ipilimumab and two cycles of platinum-based chemotherapy as first-line treatment in patients with BMs at baseline. Furthermore, several studies are exploring the combination of ICIs and radiation therapy and the activity of ICIs in patients with active BMs.

Based on the results illustrated in this review, a future effort should be made to promote real-world international studies with the aim of including a non-selected and heterogeneous population of patients with NSCLC and CNS disease. Another critical challenge is to define the best management of patients treated with ICIs and intracranial oligoprogression [76], and the best timing for multimodal treatment of patients with BM at baseline [62]. A list of ongoing clinical trials is reported in Table 3.

## 5. Conclusions

As accumulating evidence shows, immunotherapy-based combination therapy increases survival outcomes of patients with NSCLC and BMs, but how to treat active and not pre-treated lesions remains an unmet clinical need. Indeed, patients with symptomatic BMs are often excluded from clinical trials because of an estimated poor life expectancy and the risk of side effects. Moreover, international guidelines do not provide specific indications for the first-line treatment of patients with NSCLC and BMs, except for locoregional treatments options. Finally, the improvement of survival outcome with ICI combination is already consolidated, but these results were derived mostly from subgroup analysis and prospective trials investigating optimal BMs management are required.

## Figures and Tables

**Table 1:** Outcomes of patients with NSCLC and BM treated with single agent anti-PD-L1/PD-1 or anti-CTLA-4 monoclonal antibodies.

Reference	Drug	N. of pts	Histology	BM Status	PD-L1	ORR	PFS	OS
Goldberg et al. [25]	Pembrolizumab	42	NSCLC	Asymptomatic +/− RT	Cohort 1: PD-L1 ≥ 1% Cohort 2: PD-L1 < 1%	Cohort 1: 19%	1.9 m	9.9 m
Mansfield et al. [26]	Pembrolizumab vs. CT	293	NSLC	Asymptomatic (pre-treated or not)	TPS ≥ 1%	TPS ≥ 50%: 33.9% vs. 14.6% TPS ≥ 1%: 26.1% vs. 18.1%	TPS ≥ 50%: 4.1 vs. 4.6 m TPS ≥ 1%: 2.3 vs. 5.2 m	TPS ≥ 50%: 19.7 vs. 9.7 m TPS ≥ 1%: 13.4 vs. 10.3 m
Sun et al. [27]	Pembrolizumab +/− CT	131	NSCLC	Asymptomatic (pre-treated or not)	Any	27.8%	9.2 m	18.0 m
Wakuda et al. [28]	Pembrolizumab	23	NSCLC	Any	TPS ≥ 50%	57%	6.5 m	21.6 m
Borghaei et al. [29]	Nivolumab vs. docetaxel	87	NSCLC	Pre-treated and asymptomatic	Any	NA	NA	7.6 vs. 6.2 m
Cortinovis et al. [30]	Nivolumab	37	Squamous NSCLC	Pre-treated and asymptomatic	NA	19%	4.9 m	5.8 m
Grossi et al. [32]	Nivolumab	409	Non-squamous NSCLC	Pre-treated and asymptomatic	NA	NA	NA	8.6 m
Crinò et al. [33]	Nivolumab	409	Non-squamous NSCLC	Pre-treated and asymptomatic	NA	17%	3 m	8.6 m
Bidoli et al. [31]	Nivolumab	38	Squamous NSCLC	Pre-treated and asymptomatic	NA	NA	5.5 m	6.5 m
Assié et al. [34]	Nivolumab	1800	NSCLC	Pre-treated and asymptomatic	NA	NA	NA	9.9 m
Debieuvre et al. [35]	Nivolumab	477	NSCLC	Pre-treated and asymptomatic	NA	NA	NA	9.7 m
Gadgeel et al. [36]	Atezolizumab vs. docetaxel	123	NSCLC	Pre-treated and asymptomatic	NA	NA	NA	16 vs. 11.9 m
Spigel et al. [37]	Atezolizumab	13	NSCLC	Pre-treated and asymptomatic	>5%	13.2%	2.5 m	6.8 m
Sezer et al. [39]	Cemiplimab vs. CT	68	NSCLC	Asymptomatic (pre-treated or not)	≥50%	NA	13/34 vs. 26/34 events	4/34 vs. 12/34 events
Hendriks et al. [40]	ICIs	255	NSCLC	Any	NA	20.6%	1.7 m	8.5 m

BM: brain metastases; N.: number; ORR: overall response rate; PFS: progression-free survival; OS: overall survival; ICI: immune checkpoints inhibitors; CT: chemotherapy; NSCLC: non-small cell lung cancer; RT: radiotherapy; NA: not available; m: months; TPS: tumor proportional score.

**Table 2:** Outcomes of patients with NSCLC and BMs treated with combination therapies.

Reference	Drug	N. of pts	Histology	BM Status	PD-L1	ORR	PFS	OS
Hellman et al. [44]	Nivolumab + ipilimumab	81	NSCLC	Pre-treated and asymptomatic	>1% and TMB > 10	NA	4.9 m	NA
Borghaei et al. [45]	Nivolumab + ipilimumab	135	NSCLC	Pre-treated and asymptomatic	Any	33%	5.4 m	17.4 m
Barlesi et al. [46]	Nivolumab + ipilimumab	44	NSCLC	Untreated and asymptomatic	Any	37%	4.2 m	NA
Powell et al. [50]	Pembrolizumab + CT	171	NSCLC	Asymptomatic	Any	54.6%	6.9 m	18.8 m
Gadgeel et al. [51]	Pembrolizumab + CT	73	NSCLC	Asymptomatic	Any	NA	1-year PFS rate: 31.7%	1-year OS rate: 65.4%
Carbone et al. [52]	Nivolumab + ipilimumab + CT	101	NSCLC	Pre-treated and asymptomatic	Any	43%	10.6 m	19.3 m
Schapira et al. [57]	Concurrent SRS + ICI	37	NSCLC	Untreated	Any	NA	NA	1-year OS rate 87.3%
Chen et al. [61]	Concurrent SRS + ICI	157	NSCLC + others	Any	Any	NA	1-year intracranial PFS rate: 88%	NA
Sugawara et al. [68]	Nivolumab + bevacizumab + CT	36	Non squamous NSCLC	Asymptomatic	Any	NA	10.5 m	NA
Nadal et al. [40]	Atezolizumab + CT	40	Non squamous NSCLC	Untreated and asymptomatic	Any	12-week intracranial ORR: 42.5%	Intracranial PFS: 7.1 m	Intracranial OS: 8.9 m

BM: brain metastases; N.: number; ORR: overall response rate; PFS: progression-free survival; OS: overall survival; ICI: immune checkpoints inhibitors; CT: chemotherapy; NSCLC: non-small cell lung cancer; SRS: stereotactic radiosurgery; NA: not available; m: months.

**Table 3:** Ongoing clinical trials in patients with NSCLC and BMs.

Identifier	Description of the Study	Phase	Status
NCT05129202	Outcomes with Immune Checkpoint Inhibitor for Patients with Non-Small-Cell Lung Cancer and Stable Brain Metastases: A Retrospective Study	-	Recruiting
NCT05012254	Nivolumab Plus Ipilimumab Plus Two Cycles of Platinum-based Chemotherapy as First-Line Treatment for Stage IV/Recurrent Non-small Cell Lung Cancer (NSCLC) Patients with Synchronous Brain Metastases	II	Recruiting
NCT04650490	A Randomized, Phase II Trial of SRS Timing with Immune Checkpoint Inhibition in Patients with Untreated Brain Metastases from Non-small Cell Lung Cancer	II	Recruiting
NCT04187872	Recurrent Brain Metastasis Immune Effects and RespOnse to Laser Interstitial ThermotHerapy (LITT) and Pembrolizumab in Combination (TORCH)	-	Recruiting
NCT02978404	A Phase II, Multi-centre Study, of Combining Radiosurgery and Nivolumab in the Treatment of Brain Metastases from Non-small Cell Lung Cancer and Renal Cell Cancer	II	Active, not recruiting
NCT02696993	Phase I/II Trial of Nivolumab with Radiation or Nivolumab and Ipilimumab with Radiation for the Treatment of Intracranial Metastases from Non-Small Cell Lung Cancer	I/II	Recruiting
NCT04835025	A Retrospective, Multicenter Case-control Study of Radiotherapy Combined with Immunotherapy for Brain Metastases of Non-small Cell Lung Cancer	-	Suspended (because of COVID-19)
NCT01454102	A Multi-arm Phase I Safety Study of Nivolumab in Combination with Gemcitabine/Cisplatin, Pemetrexed/Cisplatin, Carboplatin/Paclitaxel, Bevacizumab Maintenance, Erlotinib, Ipilimumab or as Monotherapy in Subjects with Stage IIIB/IV Non-small Cell Lung Cancer (NSCLC)	I	Completed recruitment
NCT02681549	Pembrolizumab Plus Bevacizumab for Treatment of Brain Metastases in Metastatic Melanoma or Non-small Cell Lung Cancer	II	Recruiting
NCT02886585	Pembrolizumab in Central Nervous System Metastases (NSCLC and melanoma)	II	Recruiting

## Data Availability

No new data were created or analyzed in this study. Data sharing is not applicable to this article.
